# Knowledge of Medical Practitioners about Periodontal Diseases and Its Impact on Overall Health: A Cross-sectional Study

**DOI:** 10.7759/cureus.2694

**Published:** 2018-05-28

**Authors:** Maham Abid, Farhan Javed

**Affiliations:** 1 Periodontology, CMH Lahore Medical College and Institute of Dentistry, Shami Road, Lahore Cantt., Lahore, PAK

**Keywords:** periodontal disease, systemic diseases, systemic health, medical practitioners

## Abstract

Introduction: Periodontal disease is defined as an inflammation of the gums that may result in loss of the tissues holding the teeth in its place. The signs and symptoms include red and/or swollen gums, bleeding gums, halitosis or in severe cases, loosening of teeth. Periodontal diseases act as a risk factor for several health conditions including diabetes and cardiovascular diseases and it can even result in adverse pregnancy outcomes. Therefore, in this study, we aim to assess the knowledge of medical practitioners regarding periodontal diseases and its impact on overall health.

Methods: We conducted a cross-sectional, questionnaire-based study that included 100 medical practitioners working in the Combined Military Hospital Lahore, Pakistan.

Results: In our study, 83.0% participants knew the definition of periodontal disease; 7.0% of the participants responded that they take their patient’s periodontal history; 26.0% screened their patients for gums related problems while 87.0% reported that they are comfortable in performing an oral examination. 80.0% of the participants agreed that periodontal disease can be a risk factor for diabetes; 73.0% said they believe that it can lead to adverse pregnancy outcomes; 62.0% thought that it can result in cardiovascular diseases.

Conclusion: Most of the participants in our study had a considerable amount of knowledge about periodontal diseases. However, it is important to stress the value of taking a detailed periodontal history and screening patients for periodontal problems.

## Introduction

At some point in our lives, we come across certain oral problems such as red or inflamed gums, bleeding of gums upon toothbrushing, bad breath and in rare instances, and loosening of teeth. These signs are an indication of the presence of periodontal disease. The American Dental Association defines periodontal disease as an inflammation of the gums which can result in loss of supporting tissues of the teeth [1]. These supporting tissues include gingiva, periodontal ligament, cementum, and alveolar bone, and are collectively known as periodontium. Interestingly, there are certain conditions such as type 2 diabetes, cardiovascular diseases, and negative pregnancy outcomes that are now associated with periodontal diseases [2]. More fascinating is the fact that this interrelationship goes both ways. Not only can certain systemic diseases result in periodontal problems but periodontal diseases can also act as a risk factor for several systemic conditions. Hence, it will be safe to say that both medical doctors and dentists should be aware of this interrelationship.

In the following study, we aim to determine the knowledge of medical doctors regarding periodontal disease and its impact on overall health, with particular emphasis on diabetes, cardiovascular diseases, and negative pregnancy outcomes.

## Materials and methods

We conducted a questionnaire-based, cross-sectional study, spanning over a period of two weeks; this study was carried out in the Combined Military Hospital Lahore, Pakistan. A total of 100 randomly selected medical practitioners were included in this study from various fields of medicine using the convenience sampling method. Participants included medical specialists, general physicians, and post-graduation trainees.

## Results

All the 100 medical practitioners who took part in our study were asked if they know what a periodontal disease is to which 83.0% participants responded positively. When respondents were asked whether they take their patient’s periodontal history or not, only 7.0% gave a positive response, 26.0% participants reported that they screen their patients for periodontal problems, and 87.0% stated that they are comfortable in performing a simple oral examination (Table 1).

**Table 1:** Knowledge regarding periodontal diseases

	Yes	No
Do you know what is a periodontal disease?	83.0%	17.0%
Do you take the periodontal history of your patients?	7.0%	93.0%
Do you screen your patients for periodontal problems?	26.0%	74.0%
Are you comfortable in performing an oral examination?	87.0%	13.0%

 

We then asked the participants if they believe that periodontal disease is a risk factor for diabetes, 80.0% responded 'Yes'; 73.0% stated that periodontal disease can result in negative pregnancy outcomes and 62.0% were of the opinion that periodontal disease is a risk factor for cardiovascular diseases (Table 2).

**Table 2:** Knowledge regarding the impact of periodontal diseases on overall health

Periodontal disease is a risk factor for	Yes	No
Diabetes	80.0%	20.0%
Negative pregnancy outcome	73.0%	27.0%
Cardiovascular diseases	62.0%	38.0%

## Discussion

In 1846, William Thomas Green Morton, a dentist, became the first person to use an anesthetic agent (ether) to perform a painless tooth extraction. After observing his tremendous success, a surgeon named Henry Jacob Bigelow used a similar method to painlessly remove a neck tumor. This event simply reinforces the fact that the two fields of medicine and dentistry often overlap and dentists and physicians need to work together for the overall well-being of the patients. The same holds true for periodontal diseases.

In America, 46% of the adult population suffers from periodontitis [3]. In Pakistan, no such survey has been conducted on a national level which can tell us the prevalence of periodontal diseases among its population. However, since the periodontal diseases have an impact on overall health, medical practitioners should have knowledge on this subject. Our study showed that 83.0% of the participants had a good understanding of the term periodontitis. The fact that 17.0% of the respondents were not aware of this presents an alarming situation.

Taking patient’s periodontal history and screening the patients for periodontal problems holds great importance. Our study concluded that 7.0% participants asked their patients if they have ever been diagnosed with a periodontal problem. In a study conducted in America, 18.0% of the medical trainees confirmed that they take their patient's periodontal history [4]. In the same study, 24.0% participants screened their patients for periodontal problems. This percentage was 26.0% in our study.

One might wonder why it is important to ask patients about their periodontal status. Having periodontal problems can predispose the patients to the risk of having several diseases. As mentioned earlier, diabetes, cardiovascular diseases, and even cerebral diseases are just a few of many diseases for which a periodontal disease can act as a risk factor.

When we inquired from the participants if they are comfortable in performing an oral examination, 87.0% gave a positive response. Being able to carry out oral examination holds great importance. Sign and symptoms of various diseases such as diabetes, thrombocytopenia, and leukemia first appear in the oral cavity, resulting in gingival swelling and bleeding, hence mimicking periodontitis [5].

Diabetes has a potential to induce certain periodontal changes in the oral cavity, including gingival swelling and bleeding but can a periodontal disease have its effect on diabetes? The answer is yes; periodontitis indeed has an ability to compromise the management of diabetes as was proved in the study conducted on individuals of Gila River Indian community [6]. When the respondents of our study were asked their opinion on this, 80.0% agreed. 

Another interesting fact regarding periodontitis is that it has an ability to induce negative pregnancy outcomes including pre-term labor and low birth weight. A report published in 2013 proposes two mechanisms for this. First, the direct route, where oral microorganisms and their products have potential to reach fetal-placental barrier through hematogenous route or through the genitourinary tract. Second, an indirect route, where inflammatory mediators produced by periodontal tissues can either circulate and impact fetal-placental unit or they can circulate to the liver and enhance cytokine production, again affecting the fetal-placental unit [7]. Interestingly, only 73.0% of our participants were aware of this fact.

There is also a strong relationship between periodontitis and cardiovascular diseases. It is not surprising that a wide range of bacterial pathogens and bacterial DNA has been found in atherosclerotic plaque [8]. Oral pathogens also have a potential to enter the atherosclerotic plaque through the bloodstream leading to increased inflammatory or immune responses within the atherosclerotic plaque [9]. Only 62.0% of the respondents in our research had this knowledge.

## Conclusions

The relationship between periodontal and systemic diseases has been strongly established over the years and so is the fact that medical practitioners need to have knowledge regarding not only the periodontal diseases but also about how it can impact the overall health. The participants in our study had a fair amount of knowledge on this subject. However, medical students should be better educated and encouraged to understand and diagnose periodontal problems so that they can put a greater contribution to help improve one's life.
